# Morphological abnormalities in prefrontal surface area and thalamic volume in attention deficit/hyperactivity disorder

**DOI:** 10.1016/j.pscychresns.2015.07.004

**Published:** 2015-08-30

**Authors:** Martin J. Batty, Lena Palaniyappan, Gaia Scerif, Madeleine J. Groom, Elizabeth B. Liddle, Peter F. Liddle, Chris Hollis

**Affiliations:** a Institute of Mental Health, University of Nottingham, Nottingham, UK; b University Hospitals of Leicester NHS Trust, Leicester, UK; c Department of Experimental Psychology, University of Oxford, Oxford, UK

**Keywords:** Hyperkinetic disorder, ADHD, Magnetic resonance imaging (MRI), Thalamus, Cortex

## Abstract

Although previous morphological studies have demonstrated abnormalities in prefrontal cortical thickness in children with attention deficit/hyperactivity disorder (ADHD), studies investigating cortical surface area are lacking. As the development of cortical surface is closely linked to the establishment of thalam-ocortical connections, any abnormalities in the structure of the thalamus are likely to relate to altered cortical surface area. Using a clinically well-defined sample of children with ADHD (*n*=25, 1 female) and typically developing controls (*n*=24, 1 female), we studied surface area across the cortex to determine whether children with ADHD had reduced thalamic volume that related to prefrontal cortical surface area. Relative to controls, children with ADHD had a significant reduction in thalamic volume and dorsolateral prefrontal cortical area in both hemispheres. Furthermore, children with ADHD with smaller thalamic volumes were found to have greater reductions in surface area, a pattern not evident in the control children. Our results are further evidence of reduced lateral prefrontal cortical area in ADHD. Moreover, for the first time, we have also shown a direct association between thalamic anatomy and frontal anatomy in ADHD, suggesting the pathophysiological process that alters surface area maturation is likely to be linked to the development of the thalamus.

## Highlights

• Few studies have investigated cortical surface area in ADHD.
• Thalamic volume and cortical surface area is reduced in children with ADHD.
• Morphological thalamic abnormalities are associated with reduced cortical surface area in ADHD.
• There is an association between thalamic and frontal anatomy in ADHD.

## 1 Introduction

Attention deficit/hyperactivity disorder (ADHD) is a neurodevelopmental disorder affecting around 5% of children and young people (Polanczyk et al., 2007). It is characterised by pervasive and developmentally inappropriate levels of inattention, impulsivity and hyperactivity, and is a risk factor for the development of other disorders. ADHD was originally considered as a disorder of childhood, but it is increasingly recognised that the symptoms and pathophysiology of ADHD can persist into adulthood (Cubillo et al., 2012).

ADHD is associated with subtle abnormalities in brain structure and function. One of the earliest brain areas considered in the pathophysiology of the disorder was the thalamus (Denhoff et al., 1957) due to its key role in filtering information and stimulus processing (Gaudreau and Gagnon, 2005). Morphological abnormalities of the thalamus (Ivanov et al., 2010) and thalamic volume reduction (Xia et al., 2012) have been demonstrated previously in children with ADHD. Using diffusion tensor imaging, Silk et al. (2009) observed white matter abnormalities within the thalamus in ADHD. Various methods, including functional connectivity analysis, have uncovered thalamic abnormalities in ADHD (Cao et al., 2009, Qiu et al., 2011). Other studies have focused on striatal subcortical circuits thought to subserve attention and executive functioning (Seidman et al., 2005). Morphological abnormalities have also been found across a number of cortical regions in children and adolescents with ADHD (Seidman et al., 2005) supporting the view that cortico–striato–thalamo-cortical (CSTC) loops (Castellanos et al., 2006) play a key role in the pathogenesis of ADHD.

Morphological studies that use surface based morphometric (SBM) approaches suggest that cortical surface area and thickness have developmentally distinct trajectories, with divergent genetic influences (Panizzon et al., 2009). SBM approaches that separate these properties are potentially informative in neurodevelopmental disorders with a putative genetic basis (Palaniyappan and Liddle, 2012). In ADHD, abnormalities in prefrontal cortical thickness have already been shown by several groups (Shaw et al., 2007, Batty et al., 2010), although only two studies have investigated cortical surface area (Wolosin et al., 2009, Shaw et al., 2012). Sowell et al. (2003) compared the distance from centre (anterior commissure) across the cortical surface and reported right ventral frontal and temporal reductions in ADHD. This approach reflected a reduction in radial expansion that is more related to white matter volume and thickness than tangential expansion that is reflected by surface area measurements. With an automated technique applied at a lobar level of measurement, bilateral decreases in surface area and cortical folding have been found in children with ADHD (Wolosin et al., 2009). However, this technique precludes the ability to identify the brain region that shows the most prominent surface area reduction. Longitudinal mapping across numerous points covering the entire cortical surface (vertexwise SBM), the maturational trajectory of cortical surface area and gyrification (the degree of cortical folding measured as the ratio of the surface area of’buried’ inner cortex within sulcal folds to the ‘visible’ outer cortex) has been applied in children with ADHD and typically developing controls (Shaw et al., 2012). Intriguingly, while there were no between-group differences in the trajectory of gyrification, children with ADHD showed a significant delay in attaining their peak surface area, particularly in the right prefrontal cortex, partially mirroring an earlier finding showing delayed maturation of cortical thickness (Shaw et al., 2007). Taken together, these findings suggest that a significant maturational lag affecting multiple aspects of cortical morphology might characterise the pathophysiology of ADHD, though the origins of such a deviation in the developmental trajectory are still unclear.

Early development of cortical surface is tightly linked to the establishment of thalamocortical connections (Rakic, 2009). Animal studies suggest that allocation of specific functional areas across the cortical surface depends on both the information contained in the neural plate (protomap) and the cues received from the incoming thalamic axons (protocortex) (Clowry et al., 2010). Experimental cortical lesions that affect surface area in young male animals result in thalamic volumetric reduction (Herman et al., 1997). Thus, bidirectional organising influences between the thalamus and cortical surface are crucial in the development and specialisation of the brain in fetal and early postnatal life. In this context, abnormalities in the structure of the thalamus in putative neurodevelopmental disorders are likely to be related to cortical surface changes, the location of which will help to identify the corticothalamic circuits that are dysfunctional from a very early developmental period.

Using a clinically well-defined sample of children with ADHD and typically developing controls in whom we have previously shown reduced cortical thickness (Batty et al., 2010), we studied the cortical surface area across the entire cortex in a vertexwise fashion. Further, we sought to identify thalamic volumetric abnormality and its relationship with the surface area of the entire cortex. Given the prominence of changes in the prefrontal cortex, we hypothesised that the thalamic volume change in ADHD would be related to the surface area of the prefrontal cortex.

## 2 Methods

### 2.1 Participants

#### 2.1.1 ADHD group

Participants were recruited as part of the MIDAS (Motivation Inhibition and Development in ADHD) multi-modal neuroimaging study of ADHD, reported elsewhere (Groom et al., 2010) and using similar participants to the sample described in detail in Batty et al. (2010). Briefly, right-handed children and adolescents aged 9–15 years with a DSM-IV clinical diagnosis of ADHD combined subtype (corresponding to ICD-10 hyperkinetic disorder) and with an established positive response to methylphenidate (MPH) were recruited from child psychiatry and paediatric clinics in Nottinghamshire and Lincolnshire, UK. Parents/carers completed a battery of questionnaires, including the Development and Well-Being Assessment (DAWBA; Goodman et al., 2000), Social Communications Questionnaire (SCQ; Rutter et al., 2003), Strengths and Difficulties Questionnaire (SDQ; Goodman, 2001) and Conners long form (Connors, 1997). Permission was sought to access the child’s medical records and to contact their school, and teacher versions of the DAWBA, Conners scale and SDQ were also completed. ADHD diagnosis was confirmed or overturned following a clinical consensus diagnosis meeting, which included a full review of the child’s medical history and DAWBA interview transcripts, including computer generated predictions. Neurological abnormalities (e.g,. epilepsy, closed head injury), diagnosis of Tourette syndrome, autism spectrum disorder, bipolar disorder, major depressive disorder, learning disability (operationalised as a full scale IQ<70), history of psychosis, or current use of psychotropic medication other than melatonin were exclusion criteria. Co-morbid conduct disorder and oppositional defiant disorder (ODD) were not exclusions.

#### 2.1.2 Control group

Letters detailing the study were sent to approximately 600 families of children and adolescents attending primary and secondary schools in Nottinghamshire. From the sample who volunteered to take part, a group of right-handed controls was selected, pair-wise matched for age (± 6 months), sex and socio-economic status (SES) to a member of the ADHD group. Parents completed a shortened version of the DAWBA and the same battery of questionnaires as the ADHD group. Exclusion criteria were as detailed for the ADHD group. In addition, any participants with attention scores>4 on the SDQ (Goodman, 2001) (borderline or probable attention problem) or >1SD above the mean on the Conners’ parent or teacher long form (*n*=6) were excluded.

Ethical approval was granted by the local Research Ethics Committee and Research and Development Departments of the Nottinghamshire Healthcare and Lincolnshire Partnership NHS Trusts. After a complete description of the study, written informed consent (parents) and verbal assent (children/adolescents) was obtained. Neuroimaging data were available from 54 children. A quality control inspection assessed images for gross structural abnormalities, motion and other artefacts. Data from five subjects (all controls) were not included because of motion artefacts or poor registration of their scans. In the final sample, we report data from 49 participants; 25 ADHD (age [mean±SD], 12.66±1.78 years) and 24 typically developing controls (12.93±1.62 years).

### 2.2 MRI protocol

T1-weighted (T1W) brain images in the sagittal plane were obtained with a Philips Achieva 1.5-T MRI scanner with an eight-channel SENSE head coil using a 3D Turbo Field Echo (TFE) sequence with the following parameters: 160 contiguous slices; repetition time/echo time (TR/TE)=9.9/3.7 ms; matrix size=256×256; voxel size, 1×1×1 mm. Head movement was minimised by the use of foam pads placed within the head coil.

### 2.3 Surface extraction

Cortical surfaces were reconstructed using FreeSurfer version 5.0 (Fischl et al., 1999) in accordance with the description available on-line (http://surfer.nmr.mgh.harvard.edu/). After completion of skull-stripping and intensity correction, the grey–white matter boundary for each cortical hemisphere was determined using tissue intensity and neighbourhood constraints. With the use of a deformable surface algorithm guided by the grey-cerebrospinal fluid (CSF) intensity gradient, the resulting grey-white interface was expanded to create the pial surface, followed by a spherical morphing procedure and registration based on sulcogyral alignment. The surface boundary was tessellated to generate multiple vertices (and triangles) across the whole brain. All surfaces were visually inspected following an automated topology fixation procedure, and remaining minor defects were manually corrected as recommended by the software guidelines. Automated subcortical segmentation using probabilistic information regarding the location of subcortical structures was carried out using Freesurfer v5.0, with the investigator being blind to the diagnostic group (Fischl et al., 2002). Following this procedure, the right and left thalamic volumes were extracted. Thalamic volume estimation using Freesurfer has been shown to have an impressive consistency with stereological measurement (Keller et al., 2012), while ensuring observer blindness during measurement. We chose whole thalamic volume instead of parcellating the subdivisions of thalamus, as anatomical segmentation of thalamus using structural (T1) MRI scans alone often differs from connectivity-based identification of thalamic subdivisions obtained using white matter tractography or functional connectivity (Traynor et al., 2010).

We used methods described elsewhere (Joyner et al., 2009, Palaniyappan et al., 2011) to obtain maps of vertexwise surface area for the grey matter (pial surface) of the right and left hemisphere. Each of the several thousand vertices on the pial surface generated during the tessellation procedure was assigned the average value of the surface area of the six triangles in their immediate vicinity that define a vertex. These relative areal contraction/expansion maps were smoothed with a full-width at half-maximum Gaussian kernel of 10 mm.

### 2.4 Statistical analysis

Demographic and clinical characteristics were compared using two-tailed *t*-tests for continuous variables or chi-square (*χ*^2^) tests for categorical variables between patients and controls (CTRL). Thalamic volume was examined using a General Linear Model (GLM), with hemisphere as a within-subjects factor, diagnosis as a between-subjects factor and age, and full-scale IQ as covariates. To ensure that the group differences in thalamic volume were not confounded by individual variations in total brain size, total brain volume (summed volumes of total grey matter, total white matter and cerebrospinal fluid compartment derived from Freesurfer's segmentation procedure) was also used as a covariate in the GLM. A significance level of *p*<0.05 was used for all tests. To compare the surface area between the two groups in a vertexwise fashion, we used the Query-Design-Estimate-Contrast (QDEC) statistical interface of Freesurfer v5.0. With the use of a GLM with diagnosis as a between-subjects factor, the relative surface area change was assessed separately for the right and the left hemispheric surfaces, with age and total brain volume as covariates. To study the effect of diagnosis on the relationship between thalamic volume and the cortical surface area, total thalamic volume (summed volumes of left and right thalamus) was added as a predictor variable and the effect of the interaction between diagnosis and thalamic volume was sought while adjusting for the effects of total brain volume and age. To correct for multiple vertexwise comparisons, we undertook a Monte Carlo permutation analysis (Hagler et al., 2006). Ten thousand simulations were undertaken under the null hypothesis with a threshold of *p*=0.05 for each surface anatomical measure and the size of the largest cluster was recorded for each simulation. This multiple correction procedure was applied to both group comparison and covariance estimation. The *p*-Value for each cluster in the actual data is given by the proportion of 10,000 simulated clusters that are of same or greater size.

We explored the effect of stimulant use on the structural measures using Spearman's correlation between measures of stimulant use (current mg/kg dose of stimulants) and thalamic volume and the surface area of frontal clusters. We also related the duration of stimulants use with thalamic volume and the surface area of frontal clusters. A statistical threshold of *p*=0.05 was used for all correlational analyses.

## 3 Results

### 3.1 Demographic and clinical variables

There were no significant betweein-group differences in age, sex or socio-economic status (see Table 1). However, controls had a significantly higher full scale IQ (FSIQ) score than children with ADHD (*t* (47)=3.66, *p*=0.001) and FSIQ was used as a covariate in all subsequent analyses.

### 3.2 Thalamic volumes

Children with ADHD had a significant reduction in thalamic volume (adjusted for age and total brain volume) compared with controls (*F* (1,44 )= 5.8, *p*=0.02; adjusted mean thalamic volumes in mm^3^ in ADHD: Left (SD)=7284.05(528.20), Right=7266.22 (503.75); controls: Left=7615.49(528.23), Right=7608.56 (503.13)). Both total brain volume (*F*(1,44)=29.7, *p*<0.001) and age (*F*(1,44)=9.56, *p*=0.003) were significant positive predictors in the model, with older children and children with more total brain volume having higher thalamic volume. There was no. diagnosis × hemisphere interaction. The magnitude of these effects did not change and remained significant when the female subjects were removed from the analysis. Age and brain volume did not have any group-specific effects.

### 3.3 Surface area in ADHD

**Fig. 1:**
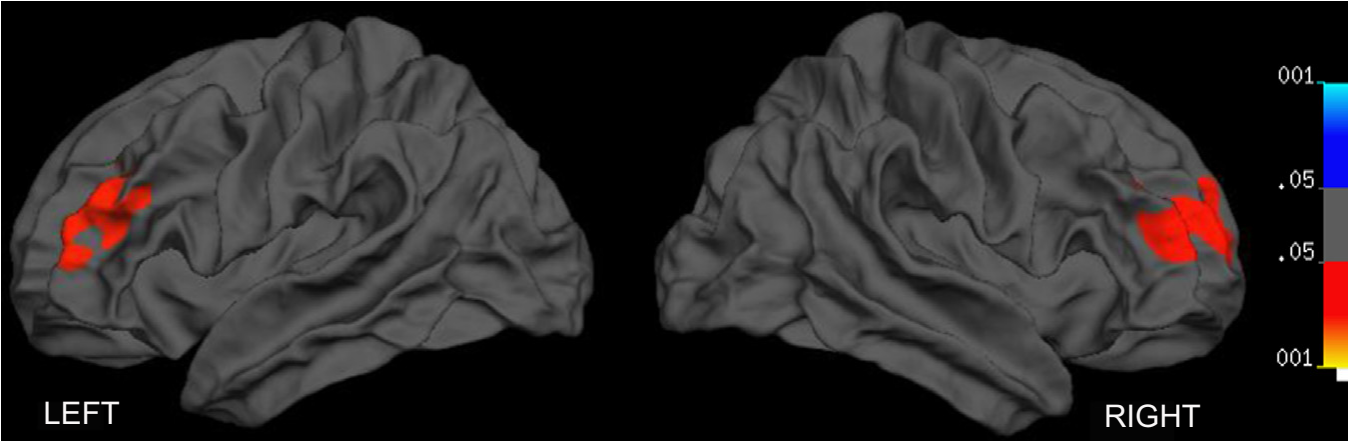
Brain regions showing significant surface area reduction in children with ADHD compared with healthy controls. No brain regions showed an increase in surface area. In this and subsequent figures, results are corrected for multiple testing using Monte Carlo permutations with an inclusion threshold of *p*=0.05. The left and right hemispheres are displayed on the left and right sides of the figure, respectively.

Vertexwise analysis of the effect of diagnosis on surface area revealed a significant reduction in dorsolateral prefrontal cortical area in children with ADHD in both right and left hemispheres. On both hemispheres, the clusters showing areal contraction overlapped with Brodmann areas (BA) 9, 10 and 46 (Fig. 1, Table 2). Those diagnostic effects remained even when the two female subjects were removed from the analysis, and when IQ was not used as a covariate. Age and brain volume did not have any significant effects on the observed differences.

### 3.4 Structural covariance between thalamus and surface area

**Fig. 2:**
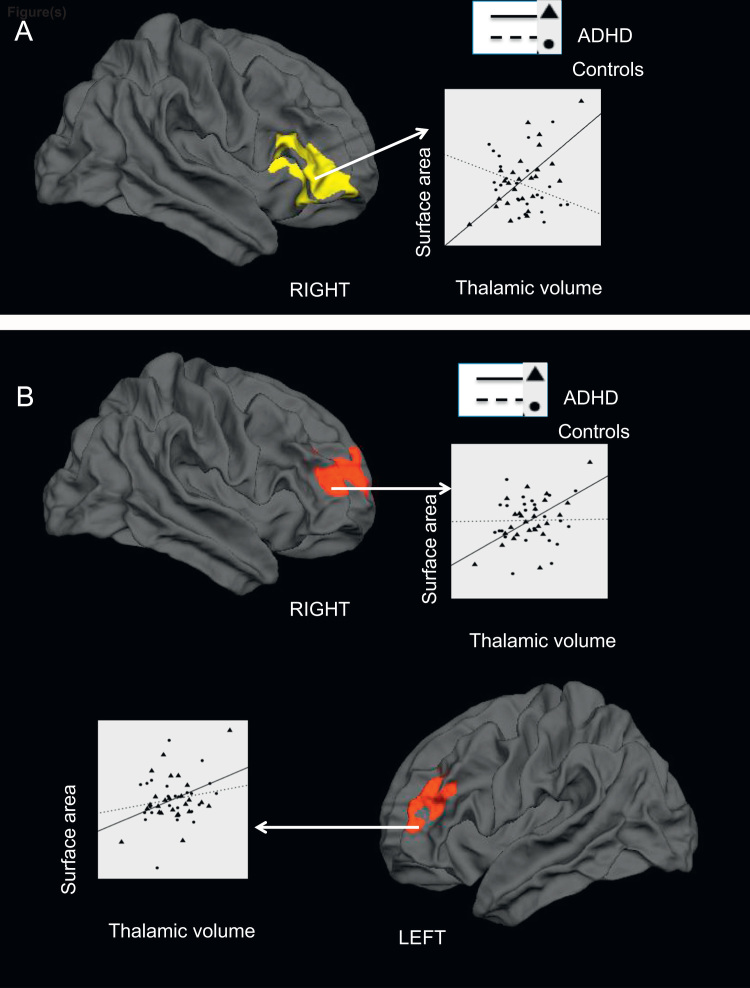
Differential effect of thalamic volume on surface area in children with ADHD and healthy controls. Panel A: A single cluster in the right prefrontal cortex showed a significant interaction between thalamic volume and diagnosis of ADHD. In this cluster, patients showed a significant positive relationship with thalamic volume, while controls showed a trend towards a negative relationship. Panel B: Scatter plots displaying the relationship between thalamic volume and surface area of right and left prefrontal clusters that showed significant surface area reduction in children with ADHD (see Fig. 1). Standardised residuals of thalamic volume and surface area measures adjusting for age and total brain volume were used for all scatter plots.

In the whole brain analysis seeking the brain regions showing differential covariance with thalamic volume in the two groups, a significant interaction between thalamic volume and the diagnosis of ADHD was seen in a cluster that included right inferior and mid-lateral prefrontal cortex (BA 10, BA 45, BA 46, BA 47; Fig. 2). In this cluster, children with ADHD with a smaller thalamic volume showed a greater reduction in the surface area (*r*=0.60, df=23, *p*=0.001), while controls showed a trend towards a negative association with thalamic volume (*r*=−0.34, df=22, *p*=0.1). No other cortical region showed an ADHD-specific relationship between thalamic volume and surface area.

**Fig. 3:**
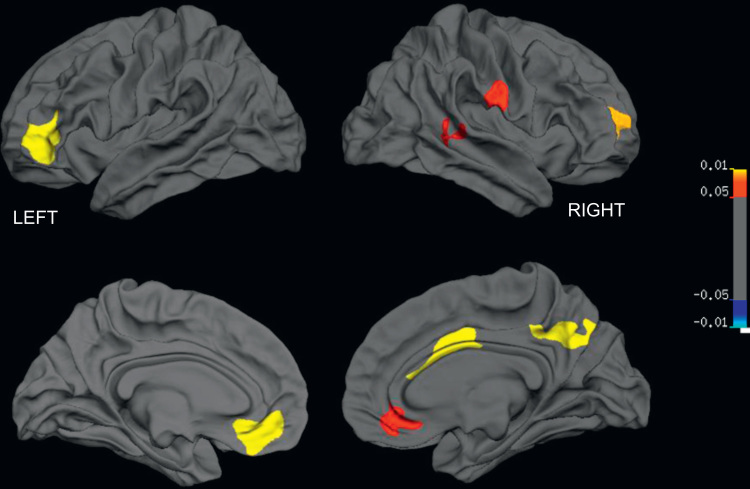
Brain regions showing significant structural covariance (positive association) with thalamic volume in children with ADHD. Lateral surfaces are shown in the top panel, medial surfaces on the bottom.

Within the patient group, thalamic volume was a positive predictor of surface area in a number of other clusters, including right precuneus, right dorsolateral frontal, and right postcentral sulcus on the right hemisphere, and left dorsolateral frontal and left anterior cingulate region on the left hemisphere (Fig. 3; Table 2). Interestingly, controls showed no such covariance with thalamic volume. These effects persisted even when IQ was not used as a covariate.

To further examine the covariance between thalamus and the bilateral frontal clusters that showed the most significant surface area reduction in ADHD, mean surface area values were extracted from the clusters for all participants and plotted against thalamic volume after adjusting for age and total brain volume. The scatter plots are shown in Fig. 2, Panel B. In children with ADHD, the right dorsolateral prefrontal cluster showed a significant positive correlation (*r*=0.59, df=23, *p*=0.002), and a trend towards a positive correlation for the left prefrontal cluster (*r*=0.39, df=23, *p*=0.052). None of these clusters showed any relationship with thalamic volume in controls (all *p*s>0.2).

Correlational analysis did not show any relationship between either the duration of stimulant use or current (mg/kg) dose of stimulants and either thalamic volume or the surface area of frontal clusters (all *p*s>0.2).

## 4 Discussion

Using a surface based morphometric approach, we have obtained further evidence of reduced lateral prefrontal cortical area in ADHD. Moreover, for the first time, we have also shown that this reduction in prefrontal cortical area covaries with smaller thalamic volume in patients with ADHD. Our findings suggest that the pathophysiological process that alters surface area maturation, especially in lateral prefrontal cortex, may well be linked to the development of the thalamus, highlighting the possibility of deviant thalamo-cortical connectivity in ADHD.

Although the functional role of the thalamus in ADHD has previously been investigated, particularly with respect to CSTC dysfunction (Dickstein et al., 2006), few structural studies have been conducted. In the first morphometric study of the thalamus in ADHD (Ivanov et al., 2010), conventional thalamic volume was not found to differ between patients and controls. However, when a more ‘fine-grained’ surface mapping approach of the thalamic surface was used, reduced bilateral volume was found in the pulvinar in young people with ADHD. In addition to its predominant connections with visual cortex and midbrain, the pulvinar region also has extensive connectivity with frontal regions (Van Essen 2005). Li et al. reported that children with ADHD, in contrast to healthy controls who show robust frontopulvinar connectivity during attentional task performance, have abnormally reduced frontopulvinar connections (Li et al., 2012). Our observation of abnormal covariance between the thalamus and the frontal cortex adds further evidence in this regard.

In contrast to the study of Ivanov et al. (2010), where gender distribution was significantly different between patients and controls, in our study, with the exception of one ADHD-control pair, all participants with ADHD were male. Consistent with our observation of reduced thalamic volume in ADHD, a multisite study using a classifier-based approach to discriminate ADHD from controls noted that subcortical structural changes were highly specific to ADHD, and the reduced grey matter intensity in thalamus highly contributed to the group membership (Colby et al., 2012). Further support for thalamic volume reduction in ADHD also comes from Xia et al. (2012), who demonstrated both volumetric reduction and shape abnormalities in the thalamus along with white matter disconnectivity involving thalamo-cortical tracts in 19 children with ADHD.

The reduction in lateral prefrontal area in ADHD has been ascribed to a developmental delay in the normal process of cortical maturation, in keeping with models that propose maturational delay as a key feature of the disorder (Shaw et al., 2012). The determinants of normal cortical maturation are at present unclear, though a number of observations suggest that the thalamus has a key role in the early development of normal cortical architecture (Kanold and Luhmann, 2010). Experimental lesions of the thalamus in young mammals can result in aberrant intracortical connectivity in later life (Kingsbury et al., 2002). In mutant mice that do not have thalamocortical connections or other extracortical guidance during development, tangential expansion (surface area formation) of the cortex is affected more than the radial extension (cortical thickness), and both the neocortex and the thalamus show significant morphometric defects (Zhou et al., 2010). Interestingly, these mutant mice also exhibit spontaneous hyperactive behaviour (Zhou et al., 2010). The covariance between thalamic volume and frontal surface area suggests the possibility that ADHD may involve pathological cortical developmental processes emerge in a period as early as the third trimester, wherein a dynamic expansion of the cortical surface area takes place (Dubois et al., 2008, Clouchoux et al., 2012). The significance of the trend level negative association between thalamus and frontal surface area in controls is at present unclear. In the absence of developmental perturbations, the frontal surface of healthy controls could continue to develop independent of the thalamic inputs, a feature that may be absent in children with ADHD.

The influence of thalamic connections on cortical maturation is likely to extend beyond the neonatal period. Preliminary evidence from diffusion tensor studies suggests that thalamocortical connectivity changes with normal development in children, with the most pronounced age-related increases in thalamocortical connectivity occurring in bilateral prefrontal areas (Alkonyi et al., 2011). Resting state functional MRI studies of thalamo-cortical connectivity in healthy children, adolescents and adults suggest that the spatial extent of the thalamo–frontal interaction develops with age (Fair et al., 2010). Taken together, these observations suggest that an aberration in thalamo-cortical connectivity in ADHD could offer a parsimonious explanation for both the frontal surface area reduction and the reduced thalamic volume evidenced in the current study.

Our cross-sectional observation is insufficient to support or refute the possibility that a reduction in the volume of the thalamus could be secondary to cortical surface changes seen in ADHD. Experimentally induced aberrations in the development of thalamo-cortical connectivity in animals result in a reduction in the volume of thalamic nuclei, in addition to a disruption in the cortical circuitry (Ghosh and Shatz, 1993, Kingsbury et al., 2002, Kanold and Luhmann, 2010). Furthermore, the present morphometric approach is not suitable to study potentially dissociable volumetric changes that may occur in individual thalamic nuclei. Thalamic nuclei have diffuse connections across the cortex, and although early primate studies postulated a specific relationship between the dorsomedial thalamus and the dorsolateral prefrontal cortex (DLPFC), a number of later observations suggest a more diffuse input from several thalamic nuclei to the lateral prefrontal region (Goldman-Rakic and Porrino, 1985, Klein et al., 2010). Imaging at a higher resolution, along with connectivity-based parcellation of the thalamus (Behrens et al., 2003), might help localise the thalamic abnormality in ADHD at a more fine-grained level.

Our observation of reduced lateral prefrontal area in ADHD is consistent with other reports (Wolosin et al., 2009, Shaw et al., 2012) despite differences in the morphometric methods used in the earlier studies. However, unlike Shaw et al. (2012), we did not observe a reduction in the surface area of the temporal lobe in the children with ADHD. This might be attributable to the limited sample size in the present study, and reinforces the fact that the lateral prefrontal cortex is the region that show the most consistent morphometric deficits in ADHD. This area is consistently implicated in the deficits noted in executive functions and cognitive control among patients with ADHD. At present, the histological underpinnings of reduced surface area are unknown, though interneurons are thought to play a crucial role in cortical surface expansion during development. The consistent localisation of reduced surface area in imaging studies of the DLPFC suggests that future studies exploring the pathological changes in brain tissue in ADHD are likely to benefit from focusing on this region.

The specific strengths of this study include a clinically well-defined sample with carefully matched controls, and the use of a well-validated surface-based automated morphometric measure that obviates the poor reliability associated with manual delineation of cortical and subcortical regions. Furthermore, we used an unbiased whole brain vertexwise approach to locate the brain region showing the most significant surface area change in ADHD. Nonetheless, there are a number of limitations that need to be considered in interpreting our results. The size of the sample is modest and, as characteristic of the disorder, was predominantly composed of males. This limits the generalisability of our findings, but it also increases the homogeneity and clinical applicability of the study. Furthermore, as is typical of the disorder, the children with ADHD had a number of comorbid disorders, particularly externalising disorders, and was underpowered to examine their specific effects. The children with ADHD also had lower IQ scores than controls. Nonetheless, the effects remained robust when IQ was covaried.

Our sample included children who were prescribed stimulant medications. Correlational analysis has not shown any relationship between either the duration of use or current dose of stimulants. Moreover, previous studies of the thalamus found that overall thalamic volume was *increased* in children with ADHD taking medication relative to unmedicated children with ADHD (Ivanov et al., 2010). As such, the observed effects of reduced thalamic volume in our study are likely if anything to be an underestimate. Nevertheless, caution is required when extrapolating our findings to unmedicated samples.

As the participants were selected on the basis of meeting diagnostic criteria for hyperkinetic disorder, the severity of ADHD symptoms in the clinical group were near ceiling, with a limited spread of symptoms. Thus, it was not possible to determine whether the severity of the morphological abnormalities was related to symptom severity. Future studies in children with a continuum of ADHD symptoms are needed to study the association between thalamo-frontal covariance and symptom profile.

In summary, our findings implicate a role for abnormal thalamo-cortical connectivity in the pathophysiology of the neurodevelopmental defects underlying ADHD. This observation will help to focus the search for genetic markers that involve very early cortical development in ADHD. Evaluating experimental approaches targeting thalamo-cortical connectivity for the effects on the frontal structural deficits associated with clinical symptoms could be an important next step in translational neuroscience of ADHD.

## Contributors

MJB and LP wrote the manuscript and carried out the statistical analysis. LP conducted the Freesurfer segmentation. All the authors read and contributed to the manuscript and the ideas presented in it. MJB and LP are joint first authors of the paper.

## Conflict of interest

LP has received a Young Investigator Travel Fellowship from Eli Lilly. In the past 5 years, PFL has received honoraria for academic presentations from Janssen-Cilag and Bristol Myers Squibb; and has taken part in advisory panels for Bristol Myers Squibb. MG has received a non-interventional ‘unrestricted’ research grant and conference support from Shire Pharmaceuticals Ltd. All other authors report no biomedical financial interests or potential conflicts of interest.

## Figures and Tables

**Table 1:** Clinical and demographic characteristics of participants.

		Group					
	ADHD (SD)		Control (SD)		Test statistic	d.f.	*p* Value
Gender	*M*=24	*F*=1	*M*=23	*F*=1	0.98	1	n/s
Age (months)	151.92	(21.31)	155.21	(19.38)	0.56	47	.575
Weight (kg)	43.96	(15.50)	51.17	(13.64)	1.73	47	.091
Medication (mg/kg)	1.07	(0.43)	0	0		47	n/a
FSIQ	91.36	(11.04)	104.79	(14.48)	3.66	47	.001
Total digit span scaled	8.12	(3.35)	9.58	(3.34)	1.53	47	.132
TOWRE: total score	88.96	(21.94)	98.33	(14.96)	1.74	47	.088
Conners Parent DSM total	82.24	(7.52)	43.83	(3.37)	23.22	47	.000
SES classification (*n*)					0.98	3	.806
Higher professional	1		1				n/s
Lower professional	5		5				n/s
Self-employed	1		0				n/s
Manual/unemployed	18		18				n/s
Co-morbid diagnoses							
ODD	12		0				n/a
CD	6		0				n/a
DCD	1		0				n/a
RD	1		0				n/a
GAD	4		0				n/a
Specific phobia	4		0				n/a
Eating disorder	1		0				n/a

*Note :* Some participants had more than one comorbidity. ADHD=attention-deficit/hyperactivity disorder; CD=conduct disorder; DCD=developmental coordination disorder; DSM=Diagnostic and Statistical Manual of Mental Disorders; FSIQ=full-scale intelligence quotient; GAD=generalised anxiety disorder; ODD=oppositional defiant disorder; RD=reading disorder; SES=socioeconomic status; TOWRE=Test of Word Reading Efficiency.

**Table 2:** Diagnostic differences in gyrification (threshold for inclusion in a cluster, *p*=0.05).

Cortical region	Talairach coordinates of the centroid (*x,y,z*)	Cluster size (mm^2^)	Clusterwise probability
Reduced surface area in patients			
Right dorsolateral prefrontal (BA 9, BA 10, BA 46)	23.6, 48.9, 15.1	1490	0.002
Left dorsolateral prefrontal (BA 9, BA 10, BA 46)	−18.4,−8.1, 54.5	1041	0.04
Increased surface area in patients None			
Structural covariance in patients			
Left dorsolateral frontal (BA 10, BA 46, BA 47)	−33.1, 50.0, 0.5	975	0.0001
Right precuneus (BA 31)	8.4, −66.6, 39.2	689	0.0001
Left medial frontal (BA 24, BA 32)	−11.3, 36.6, −9.7	617	0.0004
Right posterior cingulate (BA 24)	4.7, 1.0, 35.0	461	0.005
Right dorsolateral frontal (BA 10)	23.9, 47.1, 13.5	397	0.012
Right postcentral (BA 43)	57.9, −12.8, 27.9	338	0.033
Right medial frontal (BA 32)	11.2, 35.1, −9.5	331	0.036
Right superior temporal (BA 22)	62.7, −33.0, 7.5	317	0.044
Structural covariance in controls None			

*Note :* L BA, Brodmann area.
